# Modeling Mortality Based on Pollution and Temperature Using a New Birnbaum–Saunders Autoregressive Moving Average Structure with Regressors and Related-Sensors Data

**DOI:** 10.3390/s21196518

**Published:** 2021-09-29

**Authors:** Helton Saulo, Rubens Souza, Roberto Vila, Víctor Leiva, Robert G. Aykroyd

**Affiliations:** 1 Department of Statistics, Universidade de Brasília, Brasília 70910-90, Brazil; heltonsaulo@unb.br (H.S.); rubensbsouza@seduc.ro.gov.br (R.S.); rovig161@unb.br (R.V.); 2 School of Industrial Engineering, Pontificia Universidad Católica de Valparaíso, Valparaíso 2362807, Chile; 3 Department of Statistics, University of Leeds, Leeds, West Yorkshire LS2 9JT, UK; r.g.aykroyd@leeds.ac.uk

**Keywords:** ARMA models, Birnbaum–Saunders distribution, data dependent over time, maximum likelihood methods, model selection, Monte Carlo simulation, R software, residuals, sensing and data extraction

## Abstract

Environmental agencies are interested in relating mortality to pollutants and possible environmental contributors such as temperature. The Gaussianity assumption is often violated when modeling this relationship due to asymmetry and then other regression models should be considered. The class of Birnbaum–Saunders models, especially their regression formulations, has received considerable attention in the statistical literature. These models have been applied successfully in different areas with an emphasis on engineering, environment, and medicine. A common simplification of these models is that statistical dependence is often not considered. In this paper, we propose and derive a time-dependent model based on a reparameterized Birnbaum–Saunders (RBS) asymmetric distribution that allows us to analyze data in terms of a time-varying conditional mean. In particular, it is a dynamic class of autoregressive moving average (ARMA) models with regressors and a conditional RBS distribution (RBSARMAX). By means of a Monte Carlo simulation study, the statistical performance of the new methodology is assessed, showing good results. The asymmetric RBSARMAX structure is applied to the modeling of mortality as a function of pollution and temperature over time with sensor-related data. This modeling provides strong evidence that the new ARMA formulation is a good alternative for dealing with temporal data, particularly related to mortality with regressors of environmental temperature and pollution.

## 1. Introduction

Environmental agencies charged with establishing health-based air pollution standards are interested in determining significant relationships between pollution levels and human mortality [1]. These agencies must choose the admissible levels of these standards to protect the population including sensitive groups, such as children and the elderly, against adverse effects on their health [2]. In general, a relevant question to answer is related to the degree of association between pollutants and mortality considering possible environmental contributors, such as climate, linked mainly to temperature [3,4].

Variables associated with mortality, pollutants and temperature are often statistically related, but also their data are dependent over time. Then, a simple multiple regression is not enough to model this relationship, since a time-series structure should be considered [5]. This type of modeling is frequently conducted under the Gaussianity/normality assumption. However, such an assumption is often violated in environmental phenomena due to asymmetry and then diverse practitioners employ logarithmic transformations to reach Gaussianity. Nevertheless, data transformation brings difficulties of interpretation and power loss in statistical tests. Consequently, asymmetric models with suitable mathematical arguments for describing mortality in terms of pollution and temperature can be used. One distribution that holds with asymmetry and possesses such arguments is the Birnbaum–Saunders (BS) distribution as demonstrated in [6].

The BS distribution is a lifetime model that, in recent decades, has been widely applied in different fields of science. This distribution is continuous and unimodal, has positive asymmetry, and is supported on the set of positive real numbers. It is indexed by two parameters corresponding to its shape and scale. Proposed in [7], the BS distribution had its origins in physical problems related to a specific type of fatigue in materials under repeated stress and tension. It describes the total time until the cumulative damage caused by the development and growth of a dominant crack reaches a threshold and failure occurs. Subsequently, some assumptions made in [7] were relaxed in [8], reinforcing the physical justification for the BS model by presenting a more general derivation. For more details on the BS distribution with respect to its properties, see [9,10].

Since its first use and numerous applications in the areas of engineering and material reliability, the BS distribution family has been considered in different fields of knowledge, including environmental sciences [11,12,13,14,15,16,17,18,19]. The wide interest in this distribution is due to its theoretical arguments, its good properties, and its close relationship with the normal distribution. Several works have been performed focussing on aspects of estimation, inference, generalizations, extensions, modeling, and diagnostics in BS models. A summary of the main studies of the BS distribution can be found in [20].

In BS regressions, some forms of modeling were proposed by the authors of [21], who were the pioneers in this type of modeling. They introduced a log-linear structure for the BS distribution and developed methods for estimating parameters, hypothesis testing, and calculating confidence intervals. Later, other investigations were carried out on BS regression models such as shown and summarized in [22]. Additionally, statistical diagnostic methods were presented in [23,24] for BS models. In the same vein, diagnostic methods were formulated in [25] for BS regression models with censored observations. BS quantile regression, boundary, and bimodality have been modeled in a number of works [26,27,28,29]. A generalization of the BS distribution was derived based on elliptically contoured distributions, called the generalized BS distribution, which has been applied widely as well as its mixture [30,31]. In all of these models, the original response must be first transformed onto a logarithmic scale. This leads to a problem of interpretation of the results and to a reduction in the power of the study. In addition, although the mean ς=log(λ) is being modeled on the logarithmic scale, λ=exp(ς) is being modeled on the original scale, which, in the case of the BS distribution, corresponds to the median.

A way of dealing with the problem of logarithmic transformation usually applied in BS regression models is through reparametrization. In this sense, several reparametrizations of the BS distribution were introduced in [32], one of which, called the reparameterized BS distribution (RBS), indexes the BS distribution by its mean and precision parameters. Such a reparametrization allows the direct modeling of the mean without the need for a transformation, in a similar way to generalized linear models (GLM). Considering this mean-based RBS distribution, a GLM type regression model was introduced in [33]. In this model, the mean response is related to a linear predictor by one of the several possible link functions, and encompasses all the parameters to be estimated. Unlike all existing BS regression models, the RBS regression approach proposed in [33] allows data to be described at their original scale with ample flexibility.

Despite the growing interest in the BS distribution and the development of a considerable amount of investigation, little has been proposed for data involving a serial correlation structure. In the context of BS models, initial efforts considering a dependence structure are attributed to [34,35,36,37,38,39], and recently to [40]. As mentioned earlier, data on mortality, pollutants and temperature are often statistically related, and temporal dependence may be present. Hence, the main objective of our work is to derive a novel time-series model based on the RBS distribution, which fills a gap in a little-studied area. We derive an RBS autoregressive moving average with regressors (RBSARMAX) time-series model, which is specified in terms of a conditional mean varying over time and extends the RBS regression proposed in [33], where temporal dependence was not considered. Our approach is similar to that studied in [5,41,42]. The secondary objective is to apply the RBSARMAX structure for modeling mortality as a function of pollution and temperature with data that are related to sensors as detailed in the section on application.

The rest of this article is organized as follows. Section 2 presents the RBS distribution, some of its properties, and the RBS regression model proposed in [33]. In Section 3, the new RBSARMAX model is formulated, conditional maximum likelihood (CML) estimators of the model parameters are derived, and residual analysis is considered for this model. In Section 4, we conduct Monte Carlo simulations to evaluate the performance of the proposed methodology. Section 5 applies the RBSARMAX modeling approach to sensor-related time-series data to show its potential. The results are compared with an approach based on a Gaussian ARMA model. Finally, Section 6 provides a summary and some concluding observations, limitations, and ideas for the future of the present work.

## 2. An RBS Regression Model

### 2.1. The RBS Distribution

The RBS distribution [32], as one of the various forms of parameterization of the BS distribution, was introduced using a new parametrization of the latter as a function of its mean. The RBS distribution allows several characteristics of data modeling to be considered [32,43].

To start, if a random variable *T* follows a BS distribution, usually denoted by T∼BS(α,λ), then its cumulative distribution function (CDF) is given by:

(1) $$F_{T}\left(t;\alpha ,\lambda \right)=\Phi \left[\frac{1}{\alpha }\left(\sqrt{t/\lambda }-\sqrt{\lambda /t}\right)\right],\;t>0,\alpha >0,\lambda >0,$$

where Φ is the standard normal CDF, α is a shape parameter, and λ is a scale parameter, as well as the distribution median. Then, by considering the parameters of the BS distribution with CDF defined in (1) as $\alpha =\sqrt{2/\delta }$ and λ=μδ/(δ+1), the new parameters of the form reparametrized of the BS distribution are expressed as $\mu =\lambda (1+\alpha^{2}/2)$ and $\delta =2/\alpha^{2}$, where μ>0 is the mean of the distribution and also a scale parameter, whereas δ>0 is a shape and precision parameter. In this case, we use the notation Y∼RBS(μ,δ).

The CDF of Y∼RBS(μ,δ) is stated as:

(2) $$F\left(y;\mu ,\delta \right)=\Phi \left\{\sqrt{\frac{\delta }{2}}\left[\sqrt{\frac{(\delta +1)y}{\mu \delta }}-\sqrt{\frac{\mu \delta }{(\delta +1)y}}\;\right]\right\},\;y>0,$$

whereas the probability density function (PDF) of *Y* is obtained by differentiating the expression established in (2) with respect to *y* formulated as:

(3) $$f\left(y;\mu ,\delta \right)=\frac{\exp\left(\delta /2\right)\sqrt{\delta +1}}{4\sqrt{\pi \mu }}\;y^{-3/2}\left[y+\frac{\mu \delta }{\left(\delta +1\right)}\right]\exp\left\{-\frac{\delta }{4}\left[\frac{(\delta +1)y}{\mu \delta }+\frac{\mu \delta }{(\delta +1)y}\right]\right\},\;y>0.$$

**Figure 1:**
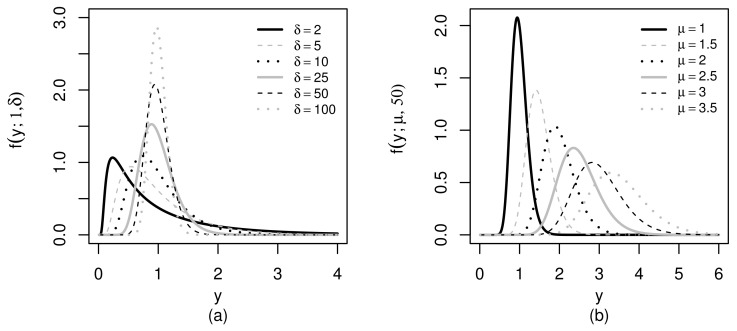
RBS(μ,δ) PDFs for μ=1 fixed (**a**) and for δ=50 fixed (**b**).

Figure 1 shows some shapes of the RBS PDF. From Figure 1a, note that δ, in addition to being a precision parameter, is also a shape parameter. Observe that, as δ increases, the PDF is more concentrated around the mean μ=1 and therefore the variability decreases. In Figure 1b, note that the distribution mean μ also behaves as a scale parameter. Hence, as it increases, there is an increase in the variance and an increased flatness in the PDF.

Due to the relationship of the BS distribution in its original version to the normal distribution, the RBS distribution has the following relationship with the normal distribution:

(4) $$Y=\frac{\mu \delta }{\delta +1}{\left[\frac{Z}{\sqrt{2\delta }}+\sqrt{{\left(\frac{Z}{\sqrt{2\delta }}\right)}^{2}+1}\right]}^{2},$$

wherein, from (4), we obtain

(5) $$Z={\left(\frac{\delta }{2}\right)}^{\frac{1}{2}}\left\{{\left[\frac{(\delta +1)Y}{\mu \delta }\right]}^{\frac{1}{2}}-{\left[\frac{\mu \delta }{(\delta +1)Y}\right]}^{\frac{1}{2}}\right\}∼N\left(0,1\right).$$

Consequently, from (4) and (5), the quantile function for the RBS distribution is expressed as:

(6) $$y\left(q;\mu ,\delta \right)=F^{-1}\left(q;\mu ,\delta \right)=\frac{\mu \delta }{\delta +1}{\left\{\frac{z(q)}{\sqrt{2\delta }}+\sqrt{{\left[\frac{z(q)}{\sqrt{2\delta }}\right]}^{2}+1}\right\}}^{2},\;0<q<1,$$

where z(q) defined in (6) is the *q*-th quantile of the standard normal distribution and $F_{Y}^{-1}$ is the inverse of the CDF of *Y* applied to *q*. The expressions for the mean and variance of the RBS distribution are stated, respectively, as:

(7) $$E\left(Y\right)=\mu ,\;\mathrm{Var}\left(Y\right)=\mu^{2}{\left[\mathrm{CV}\left(Y\right)\right]}^{2},$$

where the notation CV defined in (7) is formulated as $\mathrm{CV}\left(Y\right)=\sqrt{2\delta +5}/\left(\delta +1\right)\in \left(0,\sqrt{5}\right)$ and corresponds to the coefficient of variation of *Y*. As mentioned, δ can be interpreted as a precision parameter, that is, for fixed values of μ, when δ→∞, the variance of *Y* tends to zero. In addition, for fixed values of μ, if δ→0, then $\mathrm{Var}\left(Y\right)=5\mu^{2}$. The median of *Y* is δμ/(δ+1) and hence is proportional to the mean. Note that, for μ fixed, we have that δμ/(δ+1)→μ when δ→∞.

### 2.2. Formulation

Based on the RBS distribution, a new approach to the regression modeling of the BS distribution was proposed in [33]. In this approach, the construction of the regression model is similar to the GLM, in which the mean is directly described without the need for a transformation of the dependent variable to the logarithmic scale. Formally, consider $Y={\left(Y_{1},\cdots ,Y_{n}\right)}^{⊤}$, which is a sample of independent random variables, where each $Y_{t}∼\mathrm{RBS}\left(\mu_{t},\delta \right)$, for t∈{1,⋯,n}, and their respective observations are $y={\left(y_{1},\cdots ,y_{n}\right)}^{⊤}$. Then, a regression model based on (3) is defined by a systematic component expressed as:

(8) $$g\left(\mu_{t}\right)=\alpha_{t}=x_{t}^{⊤}\beta ,\;t\in \left\{1,\cdots ,n\right\},$$

where $x_{t}={\left(x_{t1},\cdots ,x_{tr}\right)}^{⊤}$ is a vector of known values for *r* regressors, with t∈{1,⋯,n} and r<n, $\beta ={\left(\beta_{1},\cdots ,\beta_{r}\right)}^{⊤}$ is a vector of unknown regression coefficients to be estimated, and $\alpha_{t}$ is the linear predictor. Here, we have a link function $g:R\to R^{+}$ which is strictly monotonic, always positive, and at least twice differentiable. Hence, the mean of the response variable is given by $\mu_{t}=g^{-1}\left(x_{t}^{⊤}\beta \right)$, with $g^{-1}$ being the inverse function of *g*.

### 2.3. Estimation

The logarithm of the likelihood function of the RBS regression model for the parameter vector $\gamma ={\left(\beta^{⊤},\delta \right)}^{⊤}$ has the form:

(9) $$\ell \left(\gamma \right)=\sum_{t=1}^{n}\ell_{t}\left(y_{t};\mu_{t},\delta \right),$$

where $\ell_{t}\left(y_{t};\mu_{t},\delta \right)$ defined in (9) is given by:

$$\ell_{t}\left(y_{t};\mu_{t},\delta \right)=\frac{\delta }{2}-\frac{\log(16\pi )}{2}-\frac{1}{2}\log\left[\frac{\left(\delta +1\right)y_{t}^{3}\mu_{t}}{{\left(\delta y_{t}+y_{t}+\delta \mu_{t}\right)}^{2}}\right]-\frac{\left(\delta +1\right)y_{t}}{4\mu_{t}}-\frac{\delta^{2}\mu_{t}}{4\left(\delta +1\right)y_{t}}.$$

The maximum likelihood estimate of γ is stated through solution of the system of equations $U_{\beta_{j}}\left(\gamma \right)=0$, for j∈{1,⋯,k}, and $U_{\delta }\left(\gamma \right)=0$, where $U_{\beta_{j}}\left(\gamma \right)=\partial \ell (\gamma )/\partial \beta_{j}$, and $U_{\delta }\left(\gamma \right)=\partial \ell (\gamma )/\partial \delta$. In this case, it is not possible to find an analytical solution so that the maximum likelihood estimates must be obtained numerically using an appropriate iterative method for nonlinear optimization problems, such as the Broyden–Fletcher–Goldfarb–Shanno (BFGS) quasi-Newton method, which is implemented in the R software (https://www.r-project.org, accessed on 22 September 2021) [44,45] by a command named optim.

## 3. RBSARMAX Model

### 3.1. Formulation

Let $\left\{Y_{t}\right\}$, for t∈{1,⋯,n}, be random variables such that the conditional distribution of $Y_{t}$, given the past, $F_{t-1}=\left\{Y_{t-1},\cdots ,Y_{1},\mu_{t-1},\cdots ,\mu_{1}\right\},$ follows an RBS distribution, denoted by $Y_{t}|F_{t-1}∼\mathrm{RBS}\left(\mu_{t},\delta \right)$. Then, its PDF is given by:

(10) $$f\left(y_{t};\mu_{t},\delta |F_{t-1}\right)=\frac{\exp\left(\frac{\delta }{2}\right)\sqrt{\delta +1}}{4\sqrt{\pi \mu_{t}}}y_{t}^{-3/2}\left[y_{t}+\frac{\delta \mu_{t}}{\left(\delta +1\right)}\right]\exp\left\{-\frac{\delta }{4}\left[\frac{\left(\delta +1\right)y_{t}}{\delta \mu_{t}}+\frac{\delta \mu_{t}}{\left(\delta +1\right)y_{t}}\right]\right\},\;y_{t}>0,$$

where δ>0 and $\mu_{t}=E\left[Y_{t}|F_{t-1}\right]$ are the precision parameter and the conditional mean of $Y_{t}$, respectively. Based on the RBS regression presented in (8), we postulate the RBSARMAX(p,q,r) model accommodating an additional dynamic component with an ARMA structure and regressors formulated as:

(11) $$\tau_{t}=\eta +\sum_{i=1}^{p}\phi_{i}\left[g\left(y_{t-i}\right)-x_{t-i}^{⊤}\beta \right]+\sum_{j=1}^{q}\theta_{j}\left[g\left(y_{t-j}\right)-\alpha_{t-j}\right],$$

such that now *g* defined in (11) is $g\left(\mu_{t}\right)=\alpha_{t}=x_{t}^{⊤}\beta +\tau_{t}$, for t∈{1,⋯,n}, wherein *g*, $x_{t}$, and $\beta ={\left(\beta_{1},\cdots ,\beta_{r}\right)}^{⊤}\in R^{r}$ are defined as in (8), $\phi ={\left(\phi_{1},\cdots ,\phi_{p}\right)}^{⊤}\in R^{p}$, $\theta ={\left(\theta_{1},\cdots ,\theta_{q}\right)}^{⊤}\in R^{q}$, and p,q,r∈N are the ARMAX parameters and their orders, respectively; whereas η∈R is a constant.

Therefore, we have that

(12) $$g\left(\mu_{t}\right)=\alpha_{t}=\eta +\left(x_{t}^{⊤}\beta -\sum_{i=1}^{p}\phi_{i}x_{t-i}^{⊤}\beta \right)+\sum_{i=1}^{p}\phi_{i}g\left(y_{t-i}\right)+\sum_{j=1}^{q}\theta_{j}\left[g\left(y_{t-j}\right)-\alpha_{t-j}\right].$$

The RBSARMAX model is stated by $Y_{t}|F_{t-1}∼\mathrm{RBS}\left(\mu_{t},\delta \right)$, whose PDF is defined in (10), and by the component given in (12). Note that the RBSARMAX model follows the same structure as the GARMA models [41]. For the RBSARMAX structure, the link function chosen is the identity.

### 3.2. Estimation

Parameter estimation in the RBSARMAX model is performed with the CML method or the first *m* observations, in which m=max{p,q} and n>m. From the expression stated in (10), we have that the log-likelihood function for $\gamma ={\left(\delta ,\eta ,\beta^{⊤},\phi^{⊤},\theta^{⊤}\right)}^{⊤}$ conditional on *m* observations is given by $\ell \left(\gamma \right)=\ell =\sum_{t=m+1}^{n}\ell_{t}\left(\delta ,\beta ,\eta ,\phi ,\theta \right)$, wherein $\ell_{t}\left(\delta ,\beta ,\eta ,\phi ,\theta \right)=\ell_{t}=\log\left[f\left(y_{t};\mu_{t},\delta |F_{t-1}\right)\right]$ is defined by

(13) $$\ell_{t}=\frac{\delta }{2}+\log\left[\frac{\log(16\pi )}{2}\right]-\frac{1}{2}\log\left\{\frac{\left(\delta +1\right)Y_{t}^{3}\mu_{t}}{{\left[\left(\delta +1\right)Y_{t}+\delta \mu_{t}\right]}^{2}}\right\}-\frac{Y_{t}\left(\delta +1\right)}{4\mu_{t}}-\frac{\delta^{2}\mu_{t}}{4\left(\delta +1\right)Y_{t}}.$$

The CML estimate of γ can be obtained by maximizing the log-likelihood function defined in (13), matching the score vector U(γ)=∂ℓ/∂γ to zero. Thus, the CML estimates are obtained numerically using the BFGS method. The methodology proposed in this work can be easily used by a practitioner through the R software. In particular, by employing the function garmaFit of a package named gamlss.util and some functions of the RBS package, which can be downloaded from GitHub via remotes:: install_github(“santosneto/RBS”). Note that the computational cost and complexity are relatively low. In Appendix A, we present mathematical results associated with the Fisher information matrix.

### 3.3. Residual Analysis

Residuals play a key role in the validation of any statistical model and permit us to detect the existence of outliers. In particular, two types of residuals are proposed in this study. The first is a generalized Cox–Snell (GCS) residual given by:

(14) $$r_{t}^{\mathrm{GCS}}=-\log\left[\hat{S}\left(y_{t}|F_{t-1}\right)\right],$$

wherein $\hat{S}\left(y_{t}|F_{t-1}\right)$ is the estimated survival function for the fitted model, defined as:

(15) $$\hat{S}\left(y_{t};\mu_{t},\delta \right)=\Phi \left[-{\left(\frac{\delta }{2}\right)}^{\frac{1}{2}}\left\{{\left[\frac{\left(\delta +1\right)y_{t}}{\mu_{t}\delta }\right]}^{\frac{1}{2}}-{\left[\frac{\mu_{t}\delta }{\left(\delta +1\right)y_{t}}\right]}^{\frac{1}{2}}\right\}\right],\;y_{t}>0.$$

The GCS residuals follow a unit exponential distribution, EXP (1) in short, when the model is specified correctly, and a plot of the theoretical quantiles versus empirical quantiles (QQ) of $r_{t}^{\mathrm{GCS}}$, defined in (14), can be used to assess the fit of the model to the data.

The randomized quantile (RQ) residual is also proposed, which is expressed as:

(16) $$r_{t}^{\mathrm{GS}}=\Phi^{-1}\left[\hat{S}\left(y_{t}|F_{t-1}\right)\right],$$

where $\Phi^{-1}$ is the inverse function of the CDF of the standard normal distribution and $\hat{S}\left(y_{t}|F_{t-1}\right)$ is the estimated survival function, adjusted as in (15). The RQ residual follows a standard normal distribution when the model is specified correctly. Hence, a QQ plot of the residuals defined as in (16) may be utilized to assess the fit of the model to the data.

## 4. Numerical Simulations

### 4.1. Definitions and Simulation Model

The simulations are performed using the RBSARMAX(1,1,1) model and are based on samples of size n∈{100,200,500}, considering two cases. In Case 1, simulations are performed with the values δ∈{8,15,25,50}, β=0.7, η=1.0, ϕ=0.7, and θ=0.5. For Case 2, the autoregressive (ϕ) and moving average (θ) parameters take the values of 0.3, 0.5, and 0.7, with δ=8, β=0.7, and η=1.0. These simulations evaluate the performance of the CML estimators of the RBSARMAX(1,1,1) model parameters. The simulation study is based on 1000 Monte Carlo replicates for each *n*. The proposed sample sizes aim to verify whether there are improvements in the parameter estimation as the sample size increases. The criteria used to evaluate performance for CML estimators of ϕ, θ, and δ are the empirical mean, bias, variance and mean square error (MSE) given, respectively, by:

(17) $$\bar{\hat{\varphi }}=\frac{1}{N_{r}}\sum_{r=1}^{N_{r}}{\hat{\varphi }}_{r},\;\mathrm{Bias}\left(\hat{\varphi }\right)=\bar{\hat{\varphi }}-\varphi ,\;\hat{\mathrm{Var}}\left(\hat{\varphi }\right)=\frac{1}{N_{r}}\sum_{r=1}^{N_{r}}{\left({\hat{\varphi }}_{r}-\bar{\hat{\varphi }}\right)}^{2},\;\hat{\mathrm{MSE}}\left(\hat{\varphi }\right)=\frac{1}{N_{r}}\sum_{r=1}^{N_{r}}{\left({\hat{\varphi }}_{r}-\varphi \right)}^{2},$$

where ${\hat{\varphi }}_{r}$ is the estimate obtained from the *r*-th replicate of the corresponding parameter, φ represents the true value of the parameter and $N_{r}$ is the number of Monte Carlo replicates. With the exception of the mean, for all other calculated statistics, as the value is smaller, the estimator has a better statistical performance. Note that the bias has this characteristic when analyzed in terms of its absolute value. All simulation and estimation routines were developed employing the R software.

### 4.2. RBSARMAX(1,1,1) Model

**Figure 2:**
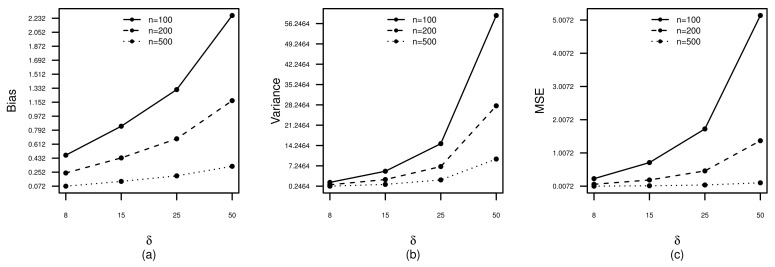
Empirical bias (**a**), variance (**b**) and MSE (**c**) of $\hat{\delta }$ with simulated data from the RBSARMAX(1,1,1) model.

Table 1 and Table 2 report the empirical mean, bias, variance, and MSE calculated as in (17) of the estimators for the shape and precision parameter (δ), autoregressive parameters (ϕ), and moving average parameters (θ), respectively. Table 1 shows the estimates for the parameter δ, fixed according to Case 1. Note that the performance of the estimator of δ is related to the sample size. For example, when the sample size increases from n=100 to n=500, the empirical bias in absolute value of the estimator of δ=8, on average, decreases considerably, from 0.4705 to 0.0720. Consequently, the mean of the estimator of δ tends to the true parameter value. In all considered scenarios, the parameter δ is, on average, overestimated, that is, the estimate $\hat{\delta }$ provided by the CML estimator for δ is greater than the true value of the parameter. The results of Table 1 are also shown in Figure 2 to simplify the interpretation of the calculated statistics in relation to the sample size and the true values of δ. Note in Figure 2a that, as n→∞, the bias of the estimator in absolute value is smaller.

The results in Table 1 and Figure 2 allow us to conclude that, in general, the performance of the estimator of δ is directly related to the sample size. That is, as n→∞, the values of the statistics are smaller and, consequently, the statistical performance of the estimator is better. Such behavior is expected, because as the sample size is greater, more information is available to estimate the parameters.

Table 2 presents summary statistics for the estimates of the parameters ϕ and θ, fixed according to the settings described for Case 2. Note that the estimators of ϕ and θ are very accurate for large sample sizes. This makes the results obtained for the MSE very close to the variance. For example, for a sample size of n=500, ϕ=0.5, and θ=0.3, the estimates are very close to the true value of the parameters, that is, $\hat{\phi }=0.4922$ and $\hat{\theta }=$ 0.3034. On average, absolute biases in estimated values of ϕ or θ are always less than 0.0336. The maximum values of the MSE are observed for ϕ=0.3 and θ=0.3 with a sample size equal to 100. Considering a fixed sample size, there is a slight reduction in the variance and MSE of the estimators of ϕ and θ as both of these parameters increase. Observe that the estimated values for ϕ and θ are, on average, underestimated. That is, the estimates $\hat{\phi }$ and $\hat{\theta }$ are less than the true parameter, in most of the considered scenarios.

### 4.3. Performance Measures and Model Selection

Performance measures are used to assess the accuracy of forecasts and compare models. These measures are a function of the observed and predicted values of the time series. Here, we consider two scenarios with respect to the data generating model: (Scenario 1) the model is correctly specified, that is, simulated values from the RBSARMAX model are generated and the RBSARMAX and Gaussian ARMA models are fitted; and (Scenario 2) the model is incorrectly specified, that is, simulated values from an ARMA model based on the Weibull distribution [46] are generated and the RBSARMAX and Gaussian ARMA models are fitted. The Weibull model was chosen because it is an asymmetrical distribution that often is considered as a competing model of the BS distribution. Then, the performance and goodness of fit of the models are compared. To evaluate the predictive ability of the models, the mean absolute percentage error (MAPE) is employed, which is given by:

(18) $$\mathrm{MAPE}=\frac{1}{n}\sum_{t=1}^{n}|\frac{\left(y_{t}-{\hat{y}}_{t}\right)}{y_{t}}|\times 100,$$

where *n* is the number of observations in the time series, $y_{t}$ is the observed value at time *t*, and ${\hat{y}}_{t}$ is the predicted value of $y_{t}$. To select the best model, we use the Akaike information criterion (AIC) and Bayesian information criterion (BIC), which are stated as:

(19) $$\begin{array}{ccc} \mathrm{AIC} & = & -2\log(L)+2k,\\ \mathrm{BIC} & = & -2\log(L)+2k\;\log(n), \end{array}$$

where *L* is the maximized likelihood for the estimated model, *n* is the number of observations, and *k* is the number of parameters. The AIC relies on the likelihood penalized by the number of model parameters, while the BIC in addition weights the number of parameters using the sample size. Smaller AIC and/or BIC values indicate better models [47].

#### 4.3.1. Scenario 1

Table 3 reports the results for sample sizes n∈{100,200,500} of the RBSARMAX(1,1,1) model, with η=1.0, β=0.7, δ=8 and ϕ,θ∈{0.3,0.5,0.7}. In the simulation, 1000 replicates are utilized for each combination of parameters. The Gaussian ARMA(1,1) model is also considered. Comparing the RBSARMAX and Gaussian ARMA estructures based on the statistics described in Table 3, note that the values of AIC and BIC highlight the fact that the RBSARMAX model fits the data better than the Gaussian ARMA model, with AIC and BIC being calculated as in (19). Considering the forecasting performance, the RBSARMAX model also provides smaller MAPE values, indicating a better forecasting capacity, with the MAPE being calculated as in (18). To measure the effects of the parameter δ on the performance of the model, Table 4 shows the summary results of 1000 Monte Carlo replicates with η=1.0, β=0.7, ϕ=0.7, θ=0.5 and δ∈{8,15,25,50}. In this case, the RBSARMAX model provides smaller values of AIC, BIC and MAPE, indicating better goodness-of-fit and forecasting ability.

#### 4.3.2. Scenario 2

Table 5 reports results for the RBSARMAX and Gaussian ARMA models. The simulated values are generated from a Weibull ARMA model with η=1.0, β=0.7 and δ=8 (shape parameter of the Weibull distribution) and ϕ,θ∈{0.3,0.5,0.7} in the case of Table 5, and from a Weibull ARMA model with η=1.0, β=0.7, ϕ=0.5, θ=0.3 and δ∈{2.5,5,8,15,25,50} in the case of Table 6. In general, the results of both tables show that the RBSARMAX model outperforms the ARMA model in terms of forecasting ability based on the MAPE and root mean squared error (RMSE), with $\mathrm{RMSE}=\sqrt{\left(1/n\right)\sum_{t=1}^{n}{\left(y_{t}-{\hat{y}}_{t}\right)}^{2}}$, where *n*, $y_{t}$ and ${\hat{y}}_{t}$ are as stated in (18). However, the selection criteria (AIC and BIC) indicate an advantage of the latter model. Since usually in time series, one is interested in forecasting, the RBSARMAX model is a better choice.

## 5. Application to Real-World Data Related to Sensors

### 5.1. Sensor-Related Data and Definition of the Variables

Next, we deal with an illustration and evaluation of the performance of the RBSARMAX model applied to a real environmental process composed of three time series related to mortality, pollutants, and temperature. Note that the pollutant data are often available from monitoring stations which are associated with sensors [48] and similarly with the temperature. On the one hand, the monitoring stations extract air from the environment for time intervals and then measure the amount of transmitted light. The measurement method is considered to be quite sensitive to particles small enough to penetrate deep into the human lung. On the other hand, the temperature sensors are electrical and electronic components that, as sensors, allow temperature to be measured using a specific electrical signal. This signal can be sent directly or by changing the resistance. They are also called heat sensors or thermosensors.

The analyzed data are available in the R software through the astsa package. These data correspond to 508 observations of weekly averages of cardiovascular mortality in Los Angeles County, CA, USA, from 1970 to 1979, associated with effects of temperature variation and levels of particulate matter (PM), which are very fine particles of solids or liquids suspended in the air [2]. The variables under analysis are mortality ($M_{t}$), temperature ($X_{1t}$) and PM ($X_{2t}$). A study similar to this was carried out in [4], which used the same dataset for regression models in the context of a time series.

### 5.2. Exploratory Data Analysis

**Figure 3:**
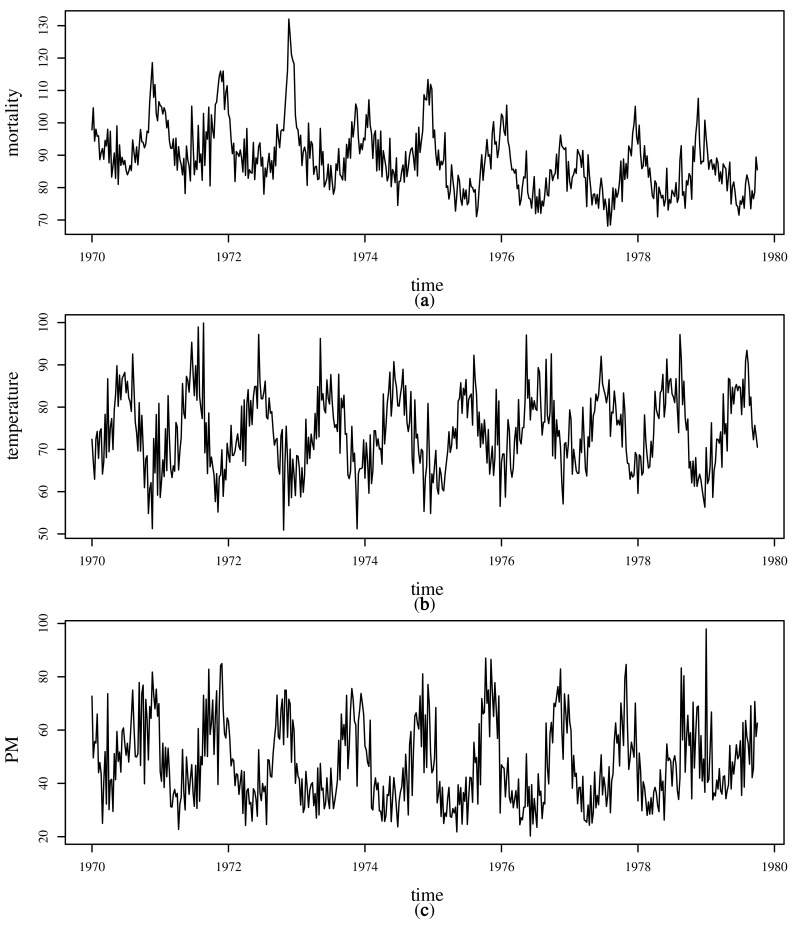
Mortality (**a**), temperature (**b**), and PM (**c**) times series over 1970–1979 in Los Angeles, CA, USA.

**Figure 4:**
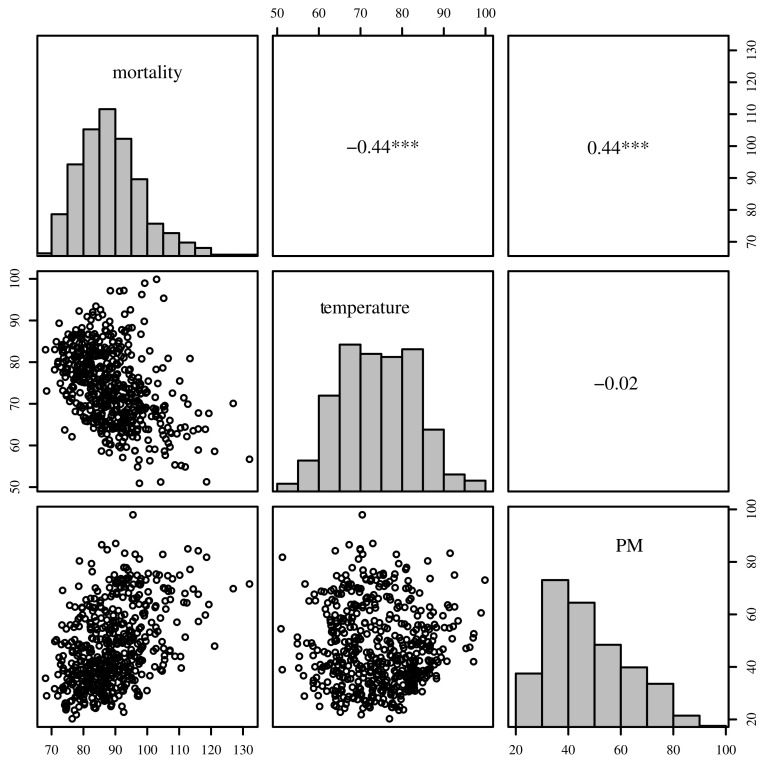
Histograms, scatterplots, and correlation coefficients of the variables: Mortality ($M_{t}$), temperature ($X_{1t}$), and PM levels ($X_{2t}$) for data over 1970–1979 in Los Angeles, CA, USA. Note that “***” indicates that such a correlation is statistically significant at 1%.

**Figure 5:**
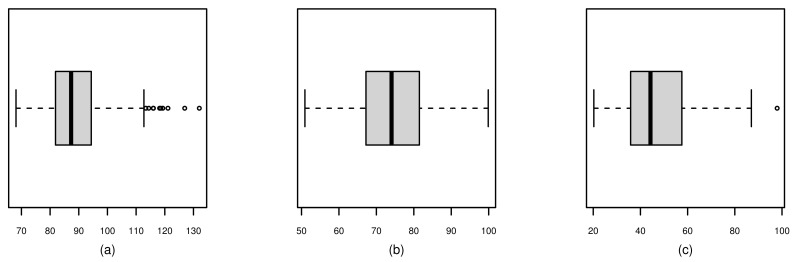
Boxplots for the variables mortality $M_{t}$ (**a**), temperature $X_{1t}$ (**b**), and PM levels $X_{2t}$ (**c**) for data over 1970–1979 in Los Angeles, CA, USA.

The behavior of the variables $M_{t}$, $X_{1t}$, and $X_{2t}$ over time are shown in Figure 3. Note that all series have a notorious seasonality. In addition, Figure 3a shows a downward trend in mortality over the period under study. Table 7 provides some descriptive measures for each variable, which include: sample size (*n*), minimum and maximum values, median, standard deviation (SD), CV, and coefficients of symmetry (CS) and kurtosis (CK). Figure 4 displays summaries of $M_{t}$, $X_{1t}$, and $X_{2t}$. Histograms are shown along the diagonal; below the diagonal are scatterplots and above the diagonal are the Pearson correlation coefficients (ρ). These graphical plots allow us to identify that mortality $M_{t}$ and temperature $X_{1t}$ have a clear relationship, with lower temperatures giving higher mortality, and that the mortality is the highest at lower temperatures. Here, $\hat{\rho }=-0.44$ indicates a moderate negative correlation which is statistically different from zero at 1% significance. Similarly, mortality $M_{t}$ and PM levels $X_{2t}$ have a linear relationship and a moderate positive correlation ($\hat{\rho }=0.44$, which is also statistically different from zero at 1% significance), indicating that higher levels of PM are associated with higher levels of mortality. However, temperature $X_{1t}$ and PM $X_{2t}$ have practically no correlation ($\hat{\rho }=-0.02$). The histograms confirm the summaries in Table 7 show that mortality $M_{t}$ and PM levels $X_{2t}$ have positive skewed behavior, whereas temperature $X_{1t}$ is more symmetric. This behavior is confirmed by the box-plots shown in Figure 5. Additionally, in this plot, the presence of outliers for mortality $M_{t}$ and PM levels $X_{2t}$ is evident.

### 5.3. Time-Series Modeling

Based on the analysis of Figure 4, which shows the relationship between the variables $M_{t}$, $X_{1t}$, and $X_{2t}$, in addition to considering $M_{t}$ as the response variable, these relationships can be modeled over time *t* using the corresponding observed values $x_{1t}$ and $x_{2t}$ as:

(20) $$M_{t}=\eta +\beta_{1}\;t+\beta_{2}\left(x_{1t}-{\bar{x}}_{1}\right)+\beta_{3}{\left(x_{1t}-{\bar{x}}_{1}\right)}^{2}+\beta_{4}x_{2t}+\varepsilon_{t},$$

where the first two terms define a linear trend in *t*, as seen in Figure 3a; the next two terms describe a quadratic relationship with temperature and ${\bar{x}}_{1}$ being the average temperature included to avoid collinearity; the next is a linear term in PM levels; and then $\varepsilon_{t}$ is a random error or a noise process. In [4], the error consists of independent and identically distributed variables with zero mean and variance $\sigma_{\varepsilon }^{2}$, whereas an alternative approach is taken here.

**Figure 6:**
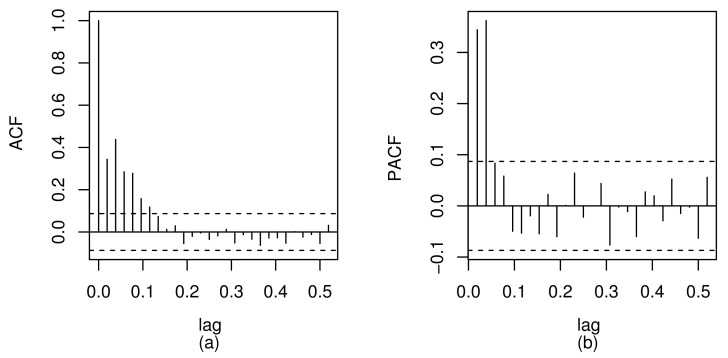
Charts of the ACF (**a**) and PACF (**b**) for the regression residuals with time-series data over the 10-year period (1970–1979) in Los Angeles County, CA, USA.

Figure 6 shows the plots of the autocorrelation function –ACF– (a) and the partial autocorrelation function –PACF– (b) of the residuals fitted with the least squares method for the model stated in (20). Consideration of the ACF and PACF plots suggests the characteristic of a stationary AR(*p*) model of order p=2 for the residuals. Thus, the correlated error model defined in (20) is expressed as: $\varepsilon_{t}=\phi_{1}\varepsilon_{t-1}+\phi_{2}\varepsilon_{t-2}+u_{t},$ where $\varepsilon_{t}$ is an AR(2) model and $u_{t}$ is a white noise. The results for this model are obtained using the garmaFit function of a package named gamlss.util (http://www.gamlss.org, accessed on 22 September 2021). Now, consider an analysis with the RBSARMAX model defined by (10) and (12). Table 8 reports the CML estimates as well as the MAPE and AIC/BIC values. From this table, note that the RBSARMAX(2,0,2) model provides a better fit than the ARMA(2,0) model based on the AIC/BIC values. Moreover, the RBSARMAX(2,0,2) model has less MAPE, indicating better forecasting capacity. We emphasize that, in addition to the advantage of these results, the RBSARMAX(2,0,2) model is more appropriate due to the skewed and kurtosis features in the data empirical distribution.

**Figure 7:**
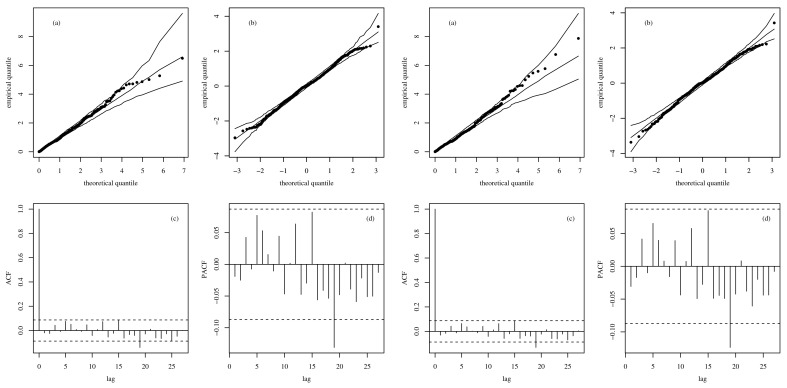
Plots of envelopes of GCS (**a**) and RQ residuals (**b**); and charts of ACF (**c**) and PACF (**d**) for the RBSARMAX (**left**) and ARMA (**right**) models.

**Figure 8:**
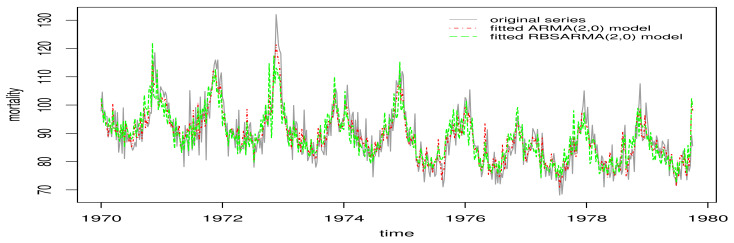
Cardiovascular mortality series for LA, USA (gray) with fitted RBSARMAX (green) and ARMA (red) models.

The QQ plots of the GCS and RQ residuals, with simulation envelopes, are presented in Figure 7a,b, respectively, which indicate better agreement with the EXP(1) distribution in the RBSARMA model. However, for the same analysis referring to the ARMA model based on Figure 7, note that the plots of GCS and RQ residuals, with simulation envelopes, produce points that are located far from the diagonal line and outside the envelope. In the ACF and PACF charts, observe that both models produce non-autocorrelated errors; see Figure 7c,d. The time-series forecasts using the fitted RBSARMAX and ARMA models are presented together with the observed time-series data in Figure 8.

## 6. Conclusions, Limitations, and Future Research

In this work, a new mean-based autoregressive moving average model using the Birnbaum–Saunders distribution, called RBSARMAX, was studied and formulated mathematically. We have estimated the model parameters with the maximum likelihood method and used information criteria for model selection to assess the adequacy of the new Birnbaum–Saunders autoregressive moving average structure.

We have conducted Monte Carlo simulations to evaluate in practice the statistical performance of the conditional maximum likelihood estimators for the parameters of the new model, showing a good performance. Additionally, in this simulation, several performance measures were used to assess the level of accuracy of forecasts and to compare different models, obtaining similarly reasonable and good results.

In the application, when modeling mortality as a function of pollution and temperature with data related to sensors, the RBSARMAX model presented a superior result to that of the Gaussian ARMA model, providing strong evidence that the Birnbaum–Saunders distribution is a good alternative for dealing with temporal data. Consequently, the results have suggested that the RBSARMAX model can become a valuable tool for analyzing positive and asymmetric time-series data in environmental sciences and other fields of knowledge.

The new methodology is an addition to the tools of applied statisticians, data scientists, and diverse users interested in the modeling of time-series data. From the application presented in this study, we have generated helpful information that may be employed by practitioners and users of statistics.

Some limitations of our proposal are described next. Since the BS distribution is related to the normal distribution, parameter estimation in RBSARMAX models may be affected by outliers and potentially influential cases. To obtain robust estimation, the BS-Student-t distribution could be considered instead [30,49]. Besides fixed effects considered by regression parameters in the RBSARMAX model, random effects may be formulated. A multivariate version of the RBSARMAX model might also be of interest [12,50], and local influence diagnostics could be derived, allowing the detection of potentially influential cases [16]. Other aspects for future study using this new model are associated with quantile, spatial, partial least squares, principal components, and sampling structures [51,52,53,54,55,56].

The authors are working on these and other aspects related to the study reported in this paper, and their findings will be presented in future articles.

## Figures and Tables

**Table 1:** CML estimates for indicated δ, based on Monte Carlo simulation of the RBSARMAX(1,1,1) model.

*n*	δ	$\hat{\delta }$			
		Mean	Bias	Variance	MSE
100	8	8.4705	0.4705	1.5462	0.2334
	15	15.8429	0.8429	5.3862	0.7171
	25	26.3139	1.3139	14.8842	1.7304
	50	52.2676	2.2676	59.0348	5.1441
200	8	8.2414	0.2414	0.7216	0.0640
	15	15.4353	0.4353	2.5264	0.1926
	25	25.6816	0.6816	7.0042	0.4665
	50	51.1722	1.1722	27.9215	1.3750
500	8	8.0720	0.0720	0.2464	0.0072
	15	15.1332	0.1332	0.8625	0.0189
	25	25.2044	0.2044	2.3963	0.0425
	50	50.3276	0.3276	9.5864	0.1077

**Table 2:** CML estimates for indicated ϕ,θ based on Monte Carlo simulation of the RBSARMAX(1,1,1) model.

*n*	ϕ	θ	$\hat{\phi }$				$\hat{\theta }$			
			Mean	Bias	Variance	MSE	Mean	Bias	Variance	MSE
100	0.3	0.3	0.2957	−0.0043	0.0258	0.0258	0.2953	−0.0047	0.0285	0.0286
		0.5	0.3087	0.0087	0.0190	0.0191	0.4791	−0.0209	0.0207	0.0211
		0.7	0.3098	0.0098	0.0139	0.0140	0.6681	−0.0319	0.0142	0.0152
	0.5	0.3	0.4761	−0.0239	0.0149	0.0155	0.3114	0.0114	0.0188	0.0189
		0.5	0.4863	−0.0137	0.0127	0.0129	0.4946	−0.0054	0.0151	0.0152
		0.7	0.4855	−0.0145	0.0110	0.0112	0.6755	−0.0245	0.0122	0.0128
	0.7	0.3	0.6664	−0.0336	0.0084	0.0096	0.3171	0.0171	0.0136	0.0139
		0.5	0.6733	−0.0267	0.0080	0.0087	0.5005	0.0005	0.0119	0.0119
		0.7	0.6725	−0.0275	0.0074	0.0081	0.6717	−0.0283	0.0109	0.0117
200	0.3	0.3	0.2954	−0.0046	0.0143	0.0143	0.2980	−0.0020	0.0151	0.0151
		0.5	0.3004	0.0004	0.0083	0.0083	0.4913	−0.0087	0.0078	0.0079
		0.7	0.3046	0.0046	0.0066	0.0066	0.6818	−0.0182	0.0052	0.0055
	0.5	0.3	0.4853	−0.0147	0.0079	0.0082	0.3066	0.0066	0.0096	0.0096
		0.5	0.4897	−0.0103	0.0054	0.0055	0.4983	−0.0017	0.0060	0.0060
		0.7	0.4920	−0.0080	0.0048	0.0049	0.6852	−0.0148	0.0045	0.0048
	0.7	0.3	0.6811	−0.0189	0.0041	0.0045	0.3093	0.0093	0.0067	0.0068
		0.5	0.6841	−0.0159	0.0033	0.0036	0.5006	0.0006	0.0050	0.0050
		0.7	0.6868	−0.0132	0.0033	0.0034	0.6817	−0.0183	0.0044	0.0047
500	0.3	0.3	0.2958	−0.0042	0.0058	0.0058	0.3002	0.0002	0.0063	0.0063
		0.5	0.2978	−0.0022	0.0036	0.0036	0.4984	−0.0016	0.0032	0.0032
		0.7	0.3018	0.0018	0.0025	0.0025	0.6922	−0.0078	0.0017	0.0018
	0.5	0.3	0.4922	−0.0078	0.0030	0.0030	0.3034	0.0034	0.0040	0.0040
		0.5	0.4936	−0.0064	0.0023	0.0024	0.5011	0.0011	0.0024	0.0024
		0.7	0.4966	−0.0034	0.0018	0.0019	0.6936	−0.0064	0.0015	0.0016
	0.7	0.3	0.6916	−0.0084	0.0014	0.0015	0.3037	0.0037	0.0029	0.0029
		0.5	0.6927	−0.0073	0.0013	0.0014	0.5013	0.0013	0.0020	0.0020
		0.7	0.6965	−0.0035	0.0013	0.0013	0.6905	−0.0095	0.0015	0.0015

**Table 3:** Forecasting comparison statistics for indicated ϕ,θ based on Monte Carlo simulations for the RBSARMAX and, in parentheses, for the ARMA model.

*n*	ϕ	θ	AIC	BIC	MAPE
100	0.3	0.3	365.1127 (424.5509)	378.1385 (437.5767)	47.5600 (50.5495)
		0.5	358.6198 (434.0864)	371.6457 (447.1122)	47.4919 (53.2613)
		0.7	353.1928 (450.4833)	366.2187 (463.5092)	47.9737 (59.0348)
	0.5	0.3	346.8663 (426.9802)	359.8921 (440.0060)	47.5695 (53.5615)
		0.5	337.8665 (441.0506)	350.8924 (454.0764)	47.5570 (58.4819)
		0.7	329.9848 (461.8964)	343.0103 (474.9222)	48.2969 (67.8709)
	0.7	0.3	304.6918 (427.5490)	317.7176 (440.5749)	47.6495 (62.3559)
		0.5	290.1231 (448.0035)	303.1489 (461.0293)	47.7313 (74.1163)
		0.7	276.5373 (474.0917)	289.5631 (487.1176)	48.8350 (94.6032)
200	0.3	0.3	730.0360 (850.0534)	746.5276 (866.5450)	48.0250 (50.4204)
		0.5	717.5611 (873.3260)	734.0527 (889.8176)	48.0832 (53.3298
		0.7	708.0656 (915.5029)	724.5571 (931.9945)	48.5205 (59.2581)
	0.5	0.3	694.7785 (858.7896)	711.2701 (875.2812)	48.0156 (53.1801)
		0.5	677.0623 (893.5079)	693.5539 (909.9995)	48.1152 (58.4859)
		0.7	662.9814 (947.2945)	679.4730 (963.7861)	48.6620 (68.0547)
	0.7	0.3	612.9718 (873.1339)	629.4634 (889.6255)	48.0465 (61.6831)
		0.5	583.2384 (924.1664)	599.7300 (940.6580)	48.2035 (74.2198)
		0.7	558.8758 (995.1106)	575.3674 (1011.6021)	48.9833 (95.2990)
500	0.3	0.3	1830.5340 (2144.955)	1851.6070 (2166.0280)	48.4820 (50.6497)
		0.5	1798.2570 (2204.2160)	1819.3300 (2225.2890)	48.5240 (53.4874)
		0.7	1768.6360 (2307.8030)	1789.7090 (2328.8760)	48.6783 (59.2412)
	0.5	0.3	1742.7360 (2176.732)	1763.8090 (2197.8050)	48.4793 (53.3932)
		0.5	1697.1640 (2265.1910)	1718.2370 (2286.2640)	48.5363 (58.5531)
		0.7	1654.8520 (2400.0260)	1675.9250 (2421.099)	48.7389 (67.9263)
	0.7	0.3	1538.2870 (2238.3820)	1559.3600 (2259.4550)	48.4948 (62.0646)
		0.5	1462.2430 (2374.6440)	1483.3160 (2395.7170)	48.5859 (74.5613)
		0.7	1391.7030 (2559.9320)	1412.7760 (2581.00500)	48.9514 (96.0765)

**Table 4:** Forecasting comparison statistics for indicated δ based on Monte Carlo simulations for the RBSARMAX and, in parentheses, for the ARMA model.

*n*	δ	AIC	BIC	MAPE
100	8	290.1231 (448.0035)	303.1489 (461.02934)	47.7313 (74.1163)
	15	282.6548 (386.9450)	295.6806 (399.9709)	32.0136 (42.2746)
	25	258.3715 (335.5085)	271.3974 (348.5343)	23.8601 (29.8148)
	50	210.3474 (265.9603)	223.3732 (278.9862)	16.4679 (20.0521)
200	8	583.2384 (924.1664)	599.7300 (940.6580)	48.2035 (74.2198)
	15	567.9856 (790.4170)	584.4772 (806.9086)	32.2836 (42.1439)
	25	518.7238 (681.6172)	535.2154 (698.1088)	24.0106 (29.5744)
	50	421.3633 (537.5253)	437.8549 (554.0169)	16.4974 (19.7077)
500	8	1462.2430 (2374.6440)	1483.3160 (2395.7170)	48.5859 (74.5613)
	15	1425.7260 (2014.7160)	1446.7990 (2035.7890)	32.4906 (42.3578)
	25	1302.9140 (1731.6710)	1323.9870 (1752.7440)	24.1287 (29.6706)
	50	1058.8260 (1363.2620)	1079.8990 (1384.3350)	16.5303 (19.6674)

**Table 5:** Forecasting comparison statistics for indicated ϕ,θ based on Monte Carlo simulations for the RBSARMAX and, in parentheses, for the ARMA model.

*n*	ϕ	θ	AIC	BIC	MAPE	RMSE
100	0.3	0.3	−22.4756 (−29.8433)	−9.4498 (−16.8175)	12.8005 (13.5334)	0.0410 (0.2342)
		0.5	−18.2110 (−24.8238)	−5.1851 (−11.7979)	13.1341 (13.9234)	0.0432 (0.2394)
		0.7	−5.8601 (−16.3406)	7.1658 (−3.3148)	14.1277 (14.5934)	0.0490 (0.2466)
	0.5	0.3	−20.0399 (−28.4447)	−7.0140 (−15.4189)	13.0553 (13.6080)	0.0428 (0.2355)
		0.5	−12.5863 (−22.6473)	0.4396 (−9.6214)	13.6356 (14.0540)	0.0465 (0.2416)
		0.7	1.2421 (−2.2657)	3.2481 (−0.2597)	2.6458 (2.7110)	0.0177 (0.0380)
	0.7	0.3	−6.4925 (−12.5952)	−0.2010 (−6.3037)	6.6156 (6.6192)	0.0229 (0.1147)
		0.5	0.1071 (−6.6168)	4.3014 (−2.4224)	4.9221 (4.6593)	0.0185 (0.0784)
		0.7	0.0340 (0.0186)	0.0600 (0.0447)	0.0447 (0.0311)	0.0000 (0.0000)
200	0.3	0.3	−48.0034 (−64.8406)	−31.5119 (−48.3490)	12.9081 (13.2018)	0.0416 (0.2191)
		0.5	−40.4854 (−57.5609)	−23.9938 (−41.0693)	13.2146 (13.4078)	0.0437 (0.2227)
		0.7	−1.7115 (−4.0864)	−0.1943 (−2.5692)	1.2999 (1.2767)	0.0045 (0.0211)
	0.5	0.3	−43.1893 (−62.3776)	−26.6977 (−45.8860)	13.1516 (13.2349)	0.0434 (0.2203)
		0.5	−17.5406 (−33.0697)	−7.4312 (−22.9603)	8.4065 (8.2611)	0.0289 (0.1378)
		0.7	−0.0234 (−0.0264)	−0.0069 (−0.0099)	0.0147 (0.0145)	0.0000 (0.0000)
	0.7	0.3	−8.0637 (−16.7478)	−3.2482 (−11.9323)	4.0371 (3.8855)	0.0140 (0.0652)
		0.5	0.1230 (−0.407)	0.2550 (−0.2751)	0.1253 (0.1108)	0.0005 (0.0018)
		0.7	−0.0036 (−0.0257)	0.0129 (−0.0092)	0.0159 (0.0148)	0.0000 (0.0000)
500	0.3	0.3	−119.9713 (−167.4259)	−98.8983 (−146.3529)	12.9788 (12.9692)	0.0423 (0.2104)
		0.5	−102.4109 (−156.7271)	−81.3378 (−135.6541)	13.2702 (13.0616)	0.0443 (0.2122)
		0.7	−667.5407 (−710.4881)	−646.4676 (−689.4151)	7.2738 (7.1659)	0.0147 (0.1301)
	0.5	0.3	−107.7683 (−162.3276)	−162.3276 (−141.2546)	13.2239 (12.9785)	0.0441 (0.0448)
		0.5	−682.3179 (−739.5599)	−661.2449 (−718.4869)	7.1669 (6.9845)	0.0143 (0.1270)
		0.7	−617.3638 (−704.0180)	−596.2907 (−682.9450)	7.6447 (7.2001)	0.0161 (0.1308)
	0.7	0.3	−680.0527 (−754.0620)	−658.9796 (−732.9890)	7.1986 (6.8827)	0.0145 (0.1259)
		0.5	−623.3151 (−733.1113)	−602.2421 (−712.0383)	7.6099 (6.9997)	0.0161 (0.1276)
		0.7	−514.9032 (−676.4386)	−494.3992 (−655.9346)	8.1062 (7.0481)	0.0183 (0.1283)

**Table 6:** Forecasting comparison statistics for indicated δ based on Monte Carlo simulations for the RBSARMAX and, in parentheses, for the ARMA model.

*n*	δ	AIC	BIC	MAPE	RMSE
100	2.5	5.1509 (5.2493)	5.5156 (5.6140)	1.6829 (1.6532)	0.0123 (0.0172)
	5	34.4187 (29.8729)	40.9968 (36.4509)	11.1338 (11.2125)	0.0530 (0.1691)
	8	−20.0399 (−28.44447)	−7.0140 (−15,4189)	13.0553 (13.6080)	0.0428 (0.2355)
	15	−139.7903 (−146.6292)	−126.7645 (−133,6034)	6.9384 (7.6037)	0.0135 (0.1662)
	25	−235.5484 (−244.4121)	−222.5225 (−231.3863)	4.3160 (4.9401)	0.0056 (0.1416)
	50	−357.0783 (−379.0394)	−344.0524 (−366.0135)	2.4303 (2.9598)	0.0021 (0.1292)
200	2.5	1.7186 (1.6450)	1.7845 (1.7110)	0.2812 (0.2697)	0.0021 (0.0027)
	5	39.4501 (33.3857)	44.2656 (38.2013)	6.5062 (6.4046)	0.0311 (0.0956)
	8	−43.1893 (−62.3776)	−26.6977 (−45.8860)	13.1516 (13.2349)	0.0434 (0.2203)
	15	−284.7475 (−299.8642)	−268.2559 (−283.3726)	6.9327 (7.1730)	0.0134 (0.1418)
	25	−477.7108 (−495,7330)	−461.2192 (−479.2414)	4.2670 (4.4941)	0.0054 (0.1113)
	50	−720.3836 (−765.6852)	−703.8920 (−749.1936)	2.3692 (2.4927)	0.0018 (0.0946)
500	2.5	2.0445 (1.9371)	2.0866 (1.9793	0.1368 (0.1246)	0.0009 (0.0013)
	5	0.7635 (0.5879)	0.8057 (0.6301)	0.0466 (0.0451)	0.0000 (0.0000)
	8	−107.7683 (−162.3276)	−86.6952 (−141.2546)	13.2239 (12.9785)	0.0441 (0.2114)
	15	−714.5877 (−758.5032)	−693.5146 (−737.4302)	6.9309 (6.8751)	0.0135 (0.1254)
	25	−1200.435 (−1248.213)	−1179.362 (−1227.139)	4.2313 (4.1990)	0.0053 (0.0886)
	50	−1811.681 (−1922.298)	−1790.608 (−1901.225)	2.3154 (2.2047)	0.0017 (0.0659)

**Table 7:** Descriptive statistics of mortality, temperature, and PM for data from Los Angeles, CA, USA.

*n*	Variables	Minimum	Maximum	Median	Mean	SD	CV	CS	CK
508	Mortality, $M_{t}$	68.110	132.040	87.330	88.699	9.999	0.113	0.804	0.981
	Temperature, $X_{1t}$	50.910	99.880	74.055	74.260	9.014	0.121	0.095	−0.459
	PM, $X_{2t}$	20.250	97.940	44.250	47.413	15.138	0.319	0.570	−0.474

**Table 8:** Parameter estimates and model adequacy for data over 1970–1979 in Los Angeles, CA, USA.

Model	Parameter	Estimate	AIC	BIC	MAPE
RBSARMAX	$\phi_{1}$	0.3646	3078.4330	3112.2770	4.8128
	$\phi_{2}$	0.4393			
	η	2842.8252			
	$\beta_{1}$	−1.3990			
	$\beta_{2}$	−0.0161			
	$\beta_{3}$	0.0154			
	$\beta_{4}$	0.1503			
	δ	623.5548			
ARMA	$\phi_{1}$	0.3881	3100.1290	3133.9730	4.8151
	$\phi_{2}$	0.4321			
	η	2831.4911			
	$\beta_{1}$	−1.3932			
	$\beta_{2}$	−0.0169			
	$\beta_{3}$	0.0154			
	$\beta_{4}$	0.1554			

## Data Availability

The data analyzed are available on request.

## Appendix A. The Observed Fisher Information Matrix

Letting $a\left(y\right)=a\left(y;\mu ,\delta \right)=\sqrt{\delta /2}\left(\sqrt{(\delta +1)y/\left(\mu \delta \right)}-\sqrt{\mu \delta /(\delta +1)y}\right)$, a simple calculation gives

(A1) $$\begin{array}{cc} \frac{\partial a(y)}{\partial y}=\frac{\sqrt{\delta }}{2\sqrt{2}y}\;\left(\sqrt{\frac{(\delta +1)y}{\mu \delta }}+\sqrt{\frac{\mu \delta }{(\delta +1)y}}\;\right)\\ \frac{\partial a(y)}{\partial \mu }=-\frac{\sqrt{\delta }}{2\sqrt{2}\mu }\;\left(\sqrt{\frac{(\delta +1)y}{\mu \delta }}+\sqrt{\frac{\mu \delta }{(\delta +1)y}}\;\right), & \frac{\partial a(y)}{\partial \delta }=-\frac{1}{2\sqrt{2}\left(\delta +1\right)\sqrt{\delta }}\;\left[\delta \left(\sqrt{\frac{\mu \delta }{(\delta +1)y}}-\sqrt{\frac{(\delta +1)y}{\mu \delta }}\;\right)+2\sqrt{\frac{\mu \delta }{(\delta +1)y}}\;\right],\\ \frac{\partial^{2}a\left(y\right)}{\partial \mu \partial y}=\frac{\sqrt{\delta }}{4\sqrt{2}\mu y}\;\left(\sqrt{\frac{\mu \delta }{(\delta +1)y}}-\sqrt{\frac{(\delta +1)y}{\mu \delta }}\;\right), & \frac{\partial^{2}a\left(y\right)}{\partial \delta \partial y}=\frac{1}{2\sqrt{2}\left(\delta +1\right)\sqrt{\delta }y}\;\left[\delta \left(\sqrt{\frac{\mu \delta }{(\delta +1)y}}+\sqrt{\frac{(\delta +1)y}{\mu \delta }}\;\right)+2\sqrt{\frac{\mu \delta }{(\delta +1)y}}\;\right],\\ \frac{\partial^{2}a\left(y\right)}{\partial \mu^{2}}=\frac{\sqrt{\delta }}{4\sqrt{2}\mu^{2}}\;\left(3\sqrt{\frac{(\delta +1)y}{\mu \delta }}+\sqrt{\frac{\mu \delta }{(\delta +1)y}}\;\right), & \frac{\partial^{3}a\left(y\right)}{\partial \mu^{2}\partial y}=-\frac{\sqrt{\delta }}{8\sqrt{2}\mu^{2}y}\;\left(\sqrt{\frac{\mu \delta }{(\delta +1)y}}-3\sqrt{\frac{(\delta +1)y}{\mu \delta }}\;\right),\\ \frac{\partial^{2}a\left(y\right)}{\partial \delta^{2}}=\frac{1}{4\sqrt{2}{\left(\delta +1\right)}^{2}\sqrt{\delta }}\;\left[\delta \left(\sqrt{\frac{\mu \delta }{(\delta +1)y}}-\sqrt{\frac{(\delta +1)y}{\mu \delta }}\;\right)+4\sqrt{\frac{\mu \delta }{(\delta +1)y}}\;\right], & \frac{\partial^{3}a\left(y\right)}{\partial \delta^{2}\partial y}=-\frac{1}{8\sqrt{2}{\left(\delta +1\right)}^{2}\sqrt{\delta }y}\;\left[\left(\delta +4\right)\sqrt{\frac{\mu \delta }{(\delta +1)y}}\;+\delta \sqrt{\frac{(\delta +1)y}{\mu \delta }}\;\right],\\ \frac{\partial^{2}a\left(y\right)}{\partial \delta \partial \mu }=-\frac{1}{4\sqrt{2}\left(\delta +1\right)\sqrt{\delta }\mu }\;\left[\delta \left(\sqrt{\frac{(\delta +1)y}{\mu \delta }}-\sqrt{\frac{\mu \delta }{(\delta +1)y}}\;\right)+2\sqrt{\frac{\mu \delta }{(\delta +1)y}}\;\right], & \frac{\partial^{3}a\left(y\right)}{\partial \delta \partial \mu \partial y}=\frac{1}{8\sqrt{2}\left(\delta +1\right)\sqrt{\delta }\mu y}\;\left[\left(\delta +2\right)\sqrt{\frac{\mu \delta }{(\delta +1)y}}\;-\delta \sqrt{\frac{(\delta +1)y}{\mu \delta }}\;\right]. \end{array}$$

Moreover, $f\left(y_{t};\mu_{t},\delta |F_{t-1}\right)=\phi \left[a\left(y_{t};\mu_{t},\delta \right)\right]\;\partial a\left(y_{t};\mu_{t},\delta \right)/\partial y_{t}$ and

$$\ell_{t}=\ell_{t}\left(\delta ,\beta ,\eta ,\phi ,\theta \right)=\log f\left(y_{t};\mu_{t},\delta |F_{t-1}\right)=\log\left\{\phi [a\left(y_{t};\mu_{t},\delta \right)]\right\}+\log\left[\frac{\partial a(y_{t};\mu_{t},\delta )}{\partial y_{t}}\right].$$

The first-order partial derivatives of $\ell_{t}$ with respect to parameters are given by:

$$\frac{\partial \ell_{t}}{\partial \delta }=-a(y_{t})\;\frac{\partial a(y_{t})}{\partial \delta }+[\frac{\partial a(y_{t})}{\partial y_{t}}{]}^{-1}\;\frac{\partial^{2}a\left(y_{t}\right)}{\partial \delta \partial y_{t}},\;\frac{\partial \ell_{t}}{\partial \gamma }=\{-a(y_{t})\;\frac{\partial a(y_{t})}{\partial \mu_{t}}+[\frac{\partial a(y_{t})}{\partial y_{t}}{]}^{-1}\;\frac{\partial^{2}a\left(y_{t}\right)}{\partial \mu_{t}\partial y_{t}}\}\frac{\partial \mu_{t}}{\partial \gamma },$$

where $\gamma \in \{\beta_{k},\eta ,\phi_{k},\theta_{l}\}$, k∈{1,⋯,p}, and l∈{1,⋯,q}. The observed Fisher information matrix is defined as: $\hat{J}\left(\delta ,\beta ,\eta ,\phi ,\theta \right)=-\partial^{2}\ell_{t}/\partial u\partial v,$ where second derivatives of $\ell_{t}$ with respect to parameters are stated as:

$$\begin{array}{cc} \; & \frac{\partial^{2}\ell_{t}}{\partial \delta^{2}}=-{\left[\frac{\partial a(y_{t})}{\partial \delta }\right]}^{2}-a(y_{t})\;\frac{\partial^{2}a\left(y_{t}\right)}{\partial \delta^{2}}-[\frac{\partial a(y_{t})}{\partial y_{t}}{]}^{-2}\;{\left[\frac{\partial^{2}a\left(y_{t}\right)}{\partial \delta \partial y_{t}}\right]}^{2}+[\frac{\partial a(y_{t})}{\partial y_{t}}{]}^{-1}\;\frac{\partial^{3}a\left(y_{t}\right)}{\partial \delta^{2}\partial y_{t}},\\ \; & \frac{\partial^{2}\ell_{t}}{\partial \gamma^{2}}=\{-a\left(y_{t}\right)\;\frac{\partial a(y_{t})}{\partial \mu_{t}}+[\frac{\partial a(y_{t})}{\partial y_{t}}{]}^{-1}\;\frac{\partial^{2}a\left(y_{t}\right)}{\partial \mu_{t}\partial y_{t}}\}\frac{\partial^{2}\mu_{t}}{\partial \gamma^{2}}\\ \; & \;+\{-{\left[\frac{\partial a(y_{t})}{\partial \mu_{t}}\right]}^{2}-a\left(y_{t}\right)\;\frac{\partial^{2}a\left(y_{t}\right)}{\partial \mu_{t}^{2}}-[\frac{\partial a(y_{t})}{\partial y_{t}}{]}^{-2}\;{\left[\frac{\partial^{2}a\left(y_{t}\right)}{\partial \mu_{t}\partial y_{t}}\right]}^{2}+[\frac{\partial a(y_{t})}{\partial y_{t}}{]}^{-1}\;\frac{\partial^{3}a\left(y_{t}\right)}{\partial \mu_{t}^{2}\partial y_{t}}\}{\left(\frac{\partial \mu_{t}}{\partial \gamma }\right)}^{2},\\ \; & \frac{\partial^{2}\ell_{t}}{\partial \delta \partial \gamma }=\{-\frac{\partial a(y_{t})}{\partial \delta }\;\frac{\partial a(y_{t})}{\partial \mu_{t}}-a\left(y_{t}\right)\;\frac{\partial^{2}a\left(y_{t}\right)}{\partial \delta \partial \mu_{t}}-[\frac{\partial a(y_{t})}{\partial y_{t}}{]}^{-2}\;\frac{\partial^{2}a\left(y_{t}\right)}{\partial \delta \partial y_{t}}\;\frac{\partial^{2}a\left(y_{t}\right)}{\partial \mu_{t}\partial y_{t}}+[\frac{\partial a(y_{t})}{\partial y_{t}}{]}^{-1}\;\frac{\partial^{3}a\left(y_{t}\right)}{\partial \mu_{t}\partial y_{t}}\}\frac{\partial \mu_{t}}{\partial \gamma },\\ \; & \frac{\partial^{2}\ell_{t}}{\partial \gamma \partial \gamma^{\prime }}=\{-a\left(y_{t}\right)\;\frac{\partial a(y_{t})}{\partial \mu_{t}}+[\frac{\partial a(y_{t})}{\partial y_{t}}{]}^{-1}\;\frac{\partial^{2}a\left(y_{t}\right)}{\partial \mu_{t}\partial y_{t}}\}\frac{\partial^{2}\mu_{t}}{\partial \gamma \partial \gamma^{\prime }}\\ \; & \;+\{-{\left[\frac{\partial a(y_{t})}{\partial \mu_{t}}\right]}^{2}-a\left(y_{t}\right)\;\frac{\partial^{2}a\left(y_{t}\right)}{\partial \mu_{t}^{2}}-[\frac{\partial a(y_{t})}{\partial y_{t}}{]}^{-2}\;\frac{\partial^{2}a\left(y_{t}\right)}{\partial \mu_{t}\partial y_{t}}\;\frac{\partial^{2}a\left(y_{t}\right)}{\partial \gamma^{\prime }\partial y_{t}}+[\frac{\partial a(y_{t})}{\partial y_{t}}{]}^{-1}\;\frac{\partial^{3}a\left(y_{t}\right)}{\partial \mu_{t}^{2}\partial y_{t}}\}\frac{\partial \mu_{t}}{\partial \gamma }\;\frac{\partial \mu_{t}}{\partial \gamma^{\prime }}, \end{array}$$

where $\gamma \in \{\beta_{k},\eta ,\phi_{k},\theta_{l}\}$ and $\gamma^{\prime }\in \left\{\beta_{r},\eta ,\phi_{r},\theta_{m}\right\}$, for k,r∈{1,⋯,p} and l,m∈{1,⋯,q}. Here, the partial derivatives of $a(y_{t})$ are presented in (A1). By using (12), the partial derivatives of $\mu_{t}$ with respect to parameters are expressed, for k∈{1,⋯,p} and l∈{1,⋯,q}, as:

$$\begin{array}{cc} \frac{\partial \mu_{t}}{\partial \eta }=\frac{1}{g^{\prime }\left(\mu_{t}\right)},\; & \frac{\partial \mu_{t}}{\partial \beta_{k}}=\frac{1}{g^{\prime }\left(\mu_{t}\right)}\;\left\{x_{tk}+\sum_{i=1}^{p}\phi_{i}\left[g\left(y_{t-i}\right)-x_{(t-i)k}\right]\right\},\\ \frac{\partial \mu_{t}}{\partial \phi_{k}}=\frac{1}{g^{\prime }\left(\mu_{t}\right)}\;\left[g\left(y_{t-k}\right)-x_{t-k}^{⊤}\beta \right],\; & \frac{\partial \mu_{t}}{\partial \theta_{l}}=\frac{1}{g^{\prime }\left(\mu_{t}\right)}\;\left[g\left(y_{t-l}\right)-\alpha_{t-l}\right],\\ \frac{\partial^{2}\mu_{t}}{\partial \gamma \partial \eta }=-\frac{g^{\prime \prime }\left(\mu_{t}\right)}{{\left[g^{\prime }\left(\mu_{t}\right)\right]}^{2}}\;\frac{\partial \mu_{t}}{\partial \gamma },\;\gamma \in \left\{\beta_{k},\eta ,\phi_{k},\theta_{l}\right\},\; & \frac{\partial^{2}\mu_{t}}{\partial \xi^{2}}=-\frac{g^{\prime \prime }\left(\mu_{t}\right)}{g^{\prime }\left(\mu_{t}\right)}\;{\left(\frac{\partial \mu_{t}}{\partial \xi }\right)}^{2},\;\xi \in \left\{\beta_{k},\phi_{k},\theta_{l}\right\},\\ \frac{\partial^{2}\mu_{t}}{\partial \theta_{l}\partial \zeta }=-\frac{g^{\prime \prime }\left(\mu_{t}\right)}{g^{\prime }\left(\mu_{t}\right)}\;\frac{\partial \mu_{t}}{\partial \theta_{l}}\;\frac{\partial \mu_{t}}{\partial \zeta },\;\zeta \in \left\{\beta_{k},\phi_{k}\right\},\; & \frac{\partial^{2}\mu_{t}}{\partial \phi_{k}\partial \beta_{k}}=-\frac{g^{\prime \prime }\left(\mu_{t}\right)}{g^{\prime }\left(\mu_{t}\right)}\;\frac{\partial \mu_{t}}{\partial \phi_{k}}\;\frac{\partial \mu_{t}}{\partial \beta_{k}}+\frac{1}{g^{\prime }\left(\mu_{t}\right)}\;\left[g\left(y_{t-k}-x_{(t-k)k}\right)\right]. \end{array}$$
